# Dietary Intake of Vitamin E and Fats Associated with Sarcopenia in Community-Dwelling Older Japanese People: A Cross-Sectional Study from the Fifth Survey of the ROAD Study

**DOI:** 10.3390/nu13051730

**Published:** 2021-05-20

**Authors:** Yuta Otsuka, Toshiko Iidaka, Chiaki Horii, Shigeyuki Muraki, Hiroyuki Oka, Kozo Nakamura, Takayuki Izumo, Tomohiro Rogi, Hiroshi Shibata, Sakae Tanaka, Noriko Yoshimura

**Affiliations:** 1 Institute for Health Care Science, Suntory Wellness Limited, 8-1-1 Seikadai, Seika-cho, Soraku-gun, Kyoto 619-0284, Japan; Takayuki_Izumo@suntory.co.jp (T.I.); Tomohiro_Rogi@suntory.co.jp (T.R.); Hiroshi_Shibata@suntory.co.jp (H.S.); 2 Department of Preventive Medicine for Locomotive Organ Disorders, 22nd Century Medical & Research Center, The University of Tokyo Hospital, Hongo 7-3-1, Bunkyo-ku, Tokyo 113-8655, Japan; iidaka-kgw@umin.ac.jp (T.I.); muraki-jsb@umin.ac.jp (S.M.); noripu@rc4.so-net.ne.jp (N.Y.); 3 Department of Orthopaedic Surgery, Sensory and Motor System Medicine, Graduate School of Medicine, The University of Tokyo, Tokyo 113-8655, Japan; harukabc@gmail.com (C.H.); tanakas-ort@h.u-tokyo.ac.jp (S.T.); 4 Department of Medical Research and Management for Musculoskeletal Pain, 22nd Century Medical and Research Center, The University of Tokyo, Tokyo 113-8655, Japan; taigis54@yahoo.co.jp; 5 Towa Hospital, Tokyo 120-0003, Japan; kozo-nakamura-62@jcom.home.ne.jp

**Keywords:** sarcopenia, ROAD study, dietary intake, vitamin E, fats

## Abstract

Dietary habits are of considerable interest as a modifiable factor for the maintenance of muscle health, especially sarcopenia. The present study aimed to investigate the association between dietary intake and sarcopenia prevalence in community-dwelling Japanese subjects. This cross-sectional study was conducted using data from the fifth survey of the Research on Osteoarthritis/Osteoporosis against Disability (ROAD) study, and 1345 participants (437 men and 908 women) aged ≥60 years were included in the analysis. Sarcopenia was determined by the definition of the Asian Working Group for Sarcopenia established in 2014, and dietary intake was assessed with the brief-type self-administered diet history questionnaire. Overall, 77 subjects (5.7%) were identified as having sarcopenia, 5.0% of men and 6.1% of women. Multiple logistic regression analysis showed that the odds ratios of sarcopenia for the dietary intake of vitamin E (α-tocopherol, 0.14 (CI 0.04–0.49), β-tocopherol (0.24, CI 0.07–0.78), γ-tocopherol (0.28, CI 0.09–0.87), and fats (fat 0.27, CI 0.08–0.96; monounsaturated fatty acids, 0.22, CI 0.07–0.72, polyunsaturated fatty acids, 0.28, CI 0.09–0.89) at the highest quantile were significantly lower compared with those at the lowest quantile. Therefore, higher dietary intakes of vitamin E and fats would be associated with a lower prevalence of sarcopenia.

## 1. Introduction

Sarcopenia is characterized by an age-related decrease in skeletal muscle mass and the loss of strength and/or physical function [1], and has become a worldwide social issue because sarcopenia induces a high risk of frailty, mobility limitation, and mortality in older populations [2]. The factors associated with sarcopenia are various and complicated, such as aging, body composition, diet, exercise habits, and inflammation status [1,3,4,5,6]. In addition to understanding its pathophysiology, many studies have focused on strategies for the prevention and treatment of sarcopenia. The importance of exercise and physical activity [5,7], as well as nutrition [4], has been well recognized as a modifiable factor for the maintenance of muscle health. There is considerable evidence that clearly shows the benefits of exercise interventions, especially resistance exercise training [8], for preventing and delaying the progression of sarcopenia in older people. However, compared to exercise interventions, the amount of evidence regarding nutrition is less, and there is a need to better understand the association of dietary factors with sarcopenia.

Several reports have shown the protective potential of nutrients for muscular health [9,10,11]. In particular, supplementary and dietary proteins play an important role in the maintenance of muscle mass and strength [9,10]. Proteins and their active metabolites, essential amino acids, exert anabolic effects of skeletal muscle synthesis via mTOR signaling [12] to enhance muscle hypertrophy [13]. In fact, a recent meta-analysis indicated that protein supplementation enhances the increase in lean body mass and hand grip strength [14,15], and high protein intake (1.0 g/kg body weight per day) was associated with better physical performance in older adults [16]. In addition to protein, the dietary intake of other nutrients such as vitamins, fatty acids, and anti-oxidants is also related to the maintenance of muscle function [9,10] through the mechanisms of anti-oxidation and anti-inflammation. However, little is known about the direct association of these nutrients with sarcopenia, the pathological condition with decreases in both muscle mass and function. Recently, Japanese cohort studies suggested that the dietary intake of protein and vitamin D [17] and the specific dietary pattern characterized by Japanese diets [18] were associated with the prevalence of sarcopenia, although a comprehensive analysis of various nutrients in a large population of community-dwelling Japanese subjects has not been documented.

The Research on Osteoarthritis/osteoporosis Against Disability (ROAD) study was a prospective cohort study which aimed to characterize the environmental and genetic backgrounds of musculoskeletal diseases such as osteoarthritis, osteoporosis, sarcopenia, and other locomotor dysfunctions in the Japanese community-dwelling population [19,20,21,22]. The ROAD study started in 2005, and the fifth survey was completed in 2018–2019. The second survey of ROAD in 2008–2010 identified the prevalence of sarcopenia using the definition of the Asian Working Group for Sarcopenia (AWGS) established in 2014 [23] as 8.5% in men and 8.0% in women among the general Japanese population ≥60 years old [21]. The same study showed that the cumulative incidences of sarcopenia increased with age in both men and women [21]. Furthermore, another study from the second ROAD survey indicated the association between the prevalence of sarcopenia and exercise habits [24], although the relationship of dietary intake with sarcopenia has not been clarified.

In the present study, data from the fifth survey of the ROAD study in which an examination of sarcopenia was conducted were examined. The aim of this study was to investigate the cross-sectional association between dietary intake and sarcopenia prevalence in community-dwelling Japanese subjects using the AWGS criteria from 2014, as well as the previous study from the second ROAD survey [21].

## 2. Materials and Methods

### 2.1. Study Participants

The data for the present study were obtained from the fifth survey of the ROAD study. The ROAD study was a prospective study of bone and joint diseases that consisted of population-based cohorts from several regions in Japan established in 2005. The details of the cohort profiles have been reported previously [19,20,21,22]. Briefly, between 2005 and 2007, a baseline database was created including clinical and genetic information for 3040 residents (1061 men and 1979 women). The subjects were recruited from resident registration lists in 3 regions from an urban region in Itabashi, Tokyo, a mountainous region in Hidakagawa, Wakayama, and a coastal region in Taiji, Wakayama.

**Figure 1:**
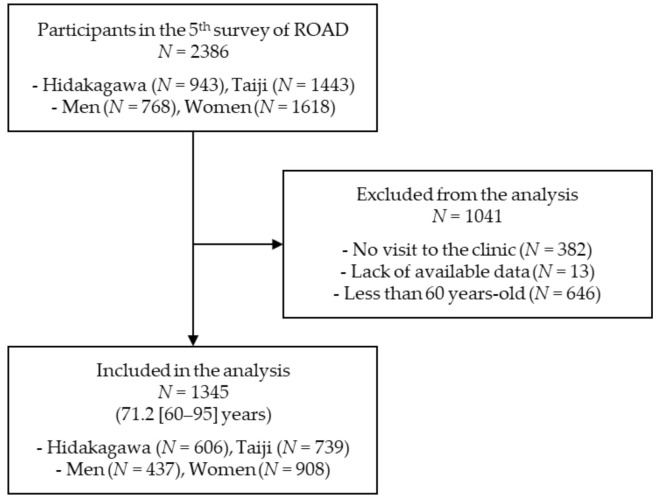
Flow chart of the recruitment of participants in this study.

The fifth survey of the ROAD study was performed only in the cohorts from the mountainous and coastal regions, from 2018 to 2019. In the fifth survey, the diagnosis of sarcopenia, including measurements of usual walking speed, grip strength, and skeletal muscle mass, was assessed, which was initiated from the second survey in 2008 [21]. In the present study, 2386 participants (768 men and 1618 women) from the mountainous and coastal regions were recruited, and participants aged ≥60 years were selected for analysis based on the AWGS criteria for sarcopenia defined in 2014 [23], as well as the second survey [21]. As described in Figure 1, 1345 (437 men and 908 women; mean age, 71.2 (standard deviation, 7.4) years [71.4 (7.9) years in men, 71.1 (7.1) years in women]) participants were included in the analysis, while 1041 participants were excluded from the analysis because of no visit to the clinic (382 participants), a lack of available data on sarcopenia assessment (13 participants), or being less than 60 years old (646 participants).

### 2.2. Interviewer-Administered Questionnaire and Anthropometric Measurements

Participants answered an interviewer-administered questionnaire, which included 200 questions concerned with lifestyle, including smoking habits and alcohol consumption. Anthropometric measurements, heights, and weights were also measured, and the body mass index (BMI; weight/height (kg/m^2^)) was calculated.

### 2.3. Walking Speed, Muscle Strength, and Skeletal Muscle Mass

For the measurement of walking speed, subjects walked 6 m at a comfortable speed, and the time taken was recorded to calculate the usual walking speed. Handgrip strength was measured with a Toei Light handgrip dynamometer (Toei Light Co. Ltd., Saitama, Japan) to evaluate muscle strength. Both hands were evaluated individually, and the largest values were used to determine the maximum grip strength. Skeletal muscle mass was measured with the Body Composition Analyzer MC-190 (Tanita Corp., Tokyo, Japan) based on bioimpedance analysis [25], whose protocol was described and validated previously [26]. Appendicular skeletal muscle mass was determined as the sum of the arms and legs’ muscle mass, which was converted to an appendicular muscle mass index by dividing by height in meters squared (kg/m^2^).

### 2.4. Definition of Sarcopenia

Sarcopenia was determined using the criteria defined by the AWGS in 2014 [23]. The cut-off values of the AWGS criteria were as follows: (A) age ≥60 or ≥65 years; (B) low appendicular skeletal muscle mass, 7.0 kg/m^2^ for men and 5.7 kg/m^2^ for women, according to bioimpedance analysis; (C) low handgrip strength, <26 kg in men and <18 kg in women; and (D) low gait speed, with a usual walking speed ≤0.8 m/s. Subjects were diagnosed as having sarcopenia if they fulfilled criteria (A), (B), and (C), or (A), (B), and (D). Regarding the age definition, not all countries use the same cut-off age to define elderly populations in the AWGS criteria due to the different states of aging in Asia. In the present study, subjects aged ≥60 years were defined as potential subjects for sarcopenia, as previously reported in the second ROAD survey [21].

### 2.5. Dietary Assessment

A self-administered brief diet history questionnaire (BDHQ), which was developed as a short version of the validated self-administered diet history questionnaire in regard to the typical Japanese diet [27] and was widely used for dietary surveys in Japan [28,29], was used. Participants completed the questionnaire at home, and it was then reviewed by well-trained interviewers in the clinic to complete all answers. The BDHQ evaluates the dietary intake frequency of 56 foods and beverage items during the past month to calculate the daily intake of energy and selected nutrients using a specific computer algorithm. In the present study, dietary intake levels of total energy and 51 major nutrient factors were analyzed.

### 2.6. Statistical Analysis

All statistical analyses were performed using JMP software, version 15.2 (SAS institute Inc, Cary, NC, USA). Differences in characteristics between men and women were compared using Student’s *t*-test for continuous variables or the chi-squared test for categorical variables. Differences in the dietary intakes of nutrient factors between participants with and without sarcopenia were compared using Student’s *t*-test. Multiple logistic regression analysis was used to evaluate the associations between the prevalence of sarcopenia and the dietary intakes of nutrient factors. In the analysis, the odds ratio (OR) and 95% confidence intervals (CI) of sarcopenia were calculated for the quintiles of the dietary intake of nutrient factors, using the lowest quintile category as the reference, after adjustment for age, sex, BMI, residence area, current smoking habit, current alcohol drinking habit, and total caloric intake. Trend associations were assessed by entering dummy variables assigned to the quintile of the dietary intake of the nutrient factors. *p* values < 0.05 were regarded as significant.

## 3. Results

### 3.1. Baseline Characteristics of the Study Population

The participants aged ≥60 years for the analysis consisted of 437 men (32.5%) and 908 women (67.5%), as shown in Table 1. There were no significant differences in age and residence area, whereas height, weight, BMI, current smoking habit, and current alcohol drinking habit were higher in men than in women. Among the indices of sarcopenia, grip strength (39.6 kg vs. 24.0 kg) and appendicular muscle mass index (7.7 kg/m^2^ vs. 6.0 kg/m^2^) were higher in men than women, but no significant difference was observed in usual walking speed (1.16 m/s vs. 1.18 m/s). In total, 77 subjects (5.7%) were identified as having sarcopenia, and there were no significant differences in the prevalence of sarcopenia between men (5.0%) and women (6.1%). According to age group stratifications of 60–64, 65–69, 70–74, 75–79, and ≥80 years, the prevalences of sarcopenia were 0.0, 0.9, 4.9, 3.8, and 25.2%, respectively (in men, 0.0, 1.7, 2.4, 2.9, and 21.1%; in women, 0.0, 0.4, 5.9, 4.1, and 27.8%, for 60–64, 65–69, 70–74, 75–79, and ≥80 years, respectively).

### 3.2. Differences between Intakes of Nutrients with and without Sarcopenia

There were no significant differences in total energy between participants with and without sarcopenia (*p* > 0.05, Table 2). However, among 51 major nutrient factors, the intakes of fat (*p* = 0.032), vegetable fat (*p* = 0.013), α-tocopherol (*p* = 0.049), β-tocopherol (*p* = 0.004), γ-tocopherol (*p* = 0.003), δ-tocopherol (*p* = 0.033), monounsaturated fatty acid (*p* = 0.016), polyunsaturated fatty acid (*p* = 0.014), and n-6 fatty acid (*p* = 0.004) were significantly lower in participants with sarcopenia than in those without sarcopenia (Table 2).

### 3.3. The Prevalence of Sarcopenia with the Amount of Vitamin E and Fats Intake

Next, the ORs of sarcopenia for quantiles of the nutrient factors for which significant differences between participants with and without sarcopenia were found were analyzed using the lowest quantile category as the reference (Table 3). The dietary intakes of α-, β-, and γ-tocopherol; fat; and monounsaturated and polyunsaturated fatty acids showed inverse associations, but δ-tocopherol, vegetable fat, and n-6 fatty acid were not significant. Adjusted for age, sex, BMI, residence area, current smoking habit, current alcohol drinking habit, and total caloric intake, the ORs for α-, β-, and γ-tocopherol at the fourth quintile were significantly lower compared with the reference values; in particular, the OR for α-tocopherol was the lowest value (OR 0.14; 95% CI 0.04–0.49), compared to β- and γ-tocopherol (OR 0.24; 95% CI 0.07–0.78; OR 0.28; 95% CI 0.09–0.87). Furthermore, among the categories of major nutrients, fat showed a significant association with a lower OR for sarcopenia (OR 0.27; 95% CI 0.08–0.96), and the ORs for monounsaturated and polyunsaturated fatty acids at the fourth quantile were also significantly lower compared with the reference value (OR 0.22; 95% CI 0.07–0.72; OR 0.28; 95% CI 0.09–0.89).

## 4. Discussion

In the present study, the prevalence of sarcopenia in the fifth survey of the ROAD study in 2018–2019 and differences in the dietary intakes of nutrient factors between participants with and without sarcopenia were examined. To the best of our knowledge, the present study is the first to demonstrate that higher dietary intakes of vitamin E and fats are associated with a low prevalence of sarcopenia in a community-dwelling older population.

The prevalence of sarcopenia in the present results was estimated using the AWGS criteria established in 2014, as well as the previous report from the second ROAD survey [21]. Compared with the second survey in 2008–2009, the prevalence in the fifth survey in 2018- 2019 was significantly lower (in the fifth survey, 5.7% [5.0% in men, 6.1% in women], Table 1; in the second survey, 8.2% [8.5% in men, 8.0% in women, *p* < 0.05]), especially in the 75–79 year age group (in the fifth survey, 0.0, 0.9, 4.9, 3.8, and 25.2%; in the second survey, 0.5, 0.0, 4.3, 11.2, and 27.0% for 60–64, 65–69, 70–74, 75–79, and ≥80 years, respectively). The reasons for these differences are related to the improvement of the physical function of elderly Japanese persons during the past decade because of increased exercise habits [30]. The prevalence was also estimated using the recently established criteria of AWGS in 2019 [31], whose value was 6.2% [5.9% in men, 6.4% in women]. However, similar trends as with AWGS2014, but no significant associations between its prevalence and the dietary intake of nutrient factors, were observed (data not shown). The cut-off values of the AWGS2014 criteria were lower for usual walking speed (≤0.8 m/s vs. <1.0 m/s) and grip strength in men (<26 kg vs. <28 kg) compared to those of AWGS2019 [31], resulting in lower statistical power for identifying significant associations among subjects with relatively mild symptoms.

Vitamin E includes four types of tocopherols (α, β, γ, and δ), whose main dietary sources are plant oils, nuts, seeds, fish, shellfish, and vegetables. Among them, α-tocopherol is the most abundant in the human body [32], and has various biological functions, such as decreasing lipid peroxide production and the maintenance of membrane fluid [33,34]. A recent review also showed the potential of vitamin E in skeletal muscle health [35], whose mechanism was related to the attenuation of oxidative stress and inflammation in age-associated muscle dysfunction [36,37,38]. In fact, some observational studies suggested that a low concentration of plasma α-tocopherol would be associated with decreased muscle strength and physical function [39,40], and the dietary intake of vitamin E was positively correlated with skeletal muscle mass [40,41], although there was no information about the relationship between its dietary intake and sarcopenia. The present study was the first to report that a higher dietary intake of vitamin E (α-, β-, and γ-tocopherol) was significantly associated with lower ORs of sarcopenia prevalence (Table 3). According to the dietary reference intake defined by the Ministry of Health, Labour and Welfare in Japan, the adequate intake levels of vitamin E, equal to α-tocopherol, are 6.5 and 6.0 mg/day in adult men and women, respectively [42], which corresponded with the values between the first and second quantiles in the present results. Therefore, higher intakes of vitamin E, rather than those of adequate levels, would prevent sarcopenia symptoms.

In the present study, there was a significant association between lower ORs for sarcopenia prevalence and higher intakes of fat, monounsaturated fatty acids, and polyunsaturated fatty acids (Table 3). The relationship between sarcopenia and fat intake has been controversial, because some previous studies have shown that a dietary pattern with a lower percentage of energy from fat was negatively related to the risk of sarcopenia [43,44], but other studies have shown that greater daily fat intake was positively related with skeletal muscle mass and lower sarcopenia prevalence [45,46]. However, the supplementation of specific fatty acids, such as omega-3 fatty acids, increases muscle mass and physical function in elderly people [47]. Furthermore, it has been reported that the plasma fatty acid profile modified by diet and exercise contributes to preventing sarcopenia [48]. Taken together, the dietary intake of fatty acids would provide a beneficial effect on sarcopenia, which is dependent on the type of fatty acid and the characteristics of the subjects. In fact, the dietary intake of oleic acid (C18:1 (M)) also showed a similar association with the OR for sarcopenia prevalence as fat in the present study (data not shown). Oleic acid, well-known as one of the omega-9 fatty acids, is contained mainly in olive oil, and has a protective role in cardiovascular disease [49]. Oleic acid has been reported to prevent muscle atrophy and increase muscle differentiation through the reduction of mitochondrial reactive oxygen species in vitro [50,51], but there have been few reports of clinical studies. Thus, further studies are needed to clarify the beneficial effect of oleic acid in sarcopenia.

The role of dietary protein in sarcopenia is well-known, and several previous studies have suggested positive associations with muscle mass and function [9,10]. However, there was no significant association between protein intake and sarcopenia prevalence in the present study (Table 2). In agreement with the present findings, some observational studies have found no association between the amounts of daily protein intake and sarcopenia or frailty, because the average daily protein intake was relatively high compared to the 0.83 g/kg BW/day recommended by the World Health Organization [52,53], and these associations would be dependent on the definition of sarcopenia [54].

There were some limitations to the present study. First, it was not possible to demonstrate a causal relationship due to the cross-sectional analysis. However, the relationship can be explained by the possibility that higher dietary intakes of vitamin E and fats may prevent the onset of sarcopenia; thus, further longitudinal analyses with 10 years of observation between the second and fifth ROAD surveys are needed to clarify these relationships in the future. Second, individuals who were able to participate in this survey from the mountainous and coastal regions may not be completely representative of the general population in Japan, although a large number of participants were enrolled in the ROAD study. Comparing the anthropometric measurements between the present study participants and the general Japanese population based on the 2018 National Health and Nutrition Survey in Japan [55], there was a significant difference in the mean BMI (22.7 (3.4) vs. 23.4 (0.6) kg/m^2^, *p* < 0.001) in subjects aged ≥60 years. Moreover, the proportion of current smokers in the present study was significantly lower in both men and women compared to the general Japanese population (men, 16.5% vs. 22.3%, *p* < 0.01; women, 2.4% vs. 4.9%, *p* < 0.01), as well as the proportion of current drinkers (men, 66.6% vs. 71.9%, *p* < 0.05; women, 28.0% vs. 35.8%, *p* < 0.001). These findings suggested that the participants in the present study had healthier lifestyles, at least regarding smoking and drinking habits, compared to the general Japanese population, leading to selection bias, which should be taken into consideration when the results in the present study are generalized. Third, the BDHQ was administered for dietary assessment, but this questionnaire has the disadvantages of low accuracy and being susceptible to seasonal effects. However, the BDHQ is considered suitable for investigating dietary habits and has been used in many cohort studies with validation by comparison with dietary records [27]. Furthermore, the strength of the ROAD study was the large population of community-dwelling subjects, and as such, the present results are considered to be highly reflective of general dietary habits.

## 5. Conclusions

In conclusion, the present study identified the associations between lower sarcopenia prevalence and higher dietary intakes of vitamin E and fats. Further longitudinal studies are needed to clarify the preventive effects of vitamin E and fats on sarcopenia.

## Figures and Tables

**Table 1:** Baseline characteristics of participants.

Characteristic	Overall (*n* = 1345)	Men (*n* = 437)	Women (*n* = 908)	*p* Value *
Age (y)	71.2 (7.4)	71.4 (7.9)	71.1 (7.1)	0.513
Height (cm)	156.1 (8.9)	165.3 (6.3)	151.7 (6.1)	**<0.001**
Weight (cm)	55.6 (10.9)	63.8 (10.8)	51.7 (8.5)	**<0.001**
BMI (kg/m^2^)	22.7 (3.4)	23.3 (3.4)	22.5 (3.4)	**<0.001**
Residing in a coastal area (%)	54.9	53.1	55.8	0.343
Current smoking habit (%)	7.0	16.5	2.4	**<0.001**
Current alcohol drinking habit (%)	40.5	66.6	28.0	**<0.001**
Usual walking speed (m/s)	1.17 (0.26)	1.16 (0.25)	1.18 (0.26)	0.140
Handgrip strength (kg)	29.1 (9.5)	39.6 (7.8)	24.0 (4.8)	**<0.001**
Appendicular muscle mass index (kg/m^2^)	6.6 (1.1)	7.7 (1.1)	6.0 (0.6)	**<0.001**
Prevalence of sarcopenia (%)	5.7	5.0	6.1	0.450

Values are means (standard deviation). BMI, body mass index. * Continuous variables, Student’s *t*-test; categorical variables, chi-squared test, and *p* < 0.05 shown as boldface.

**Table 2:** Dietary intakes of total energy and 50 major nutrient factors in participants with and without sarcopenia.

Category	Nutrient Factor	Sarcopenia (+) (*n* = 77)	Sarcopenia (−) (*n* = 1268)	*p* Value *
Calorie	Total energy (kcal/day)	1808.4 (522.2)	1877.8 (559.4)	0.289
Major nutrient	Protein (g/day)	73.3 (27.4)	75.9 (28.2)	0.433
	Animal protein (g/day)	44.8 (21.8)	46.6 (22.4)	0.493
	Vegetable protein (g/day)	28.5 (8.6)	29.3 (9.2)	0.462
	Fat (g/day)	50.6 (19.2)	55.7 (20.6)	**0.032**
	Animal fat (g/day)	25.4 (12.1)	27.3 (12.2)	0.181
	Vegetable fat (g/day)	25.2 (10.2)	28.4 (11.1)	**0.013**
	Carbohydrate (g/day)	249.6 (72.0)	247.5 (79.3)	0.823
Mineral	Ash (g/day)	18.8 (6.4)	19.6 (6.5)	0.268
	Sodium (mg/day)	4314.7 (1385.3)	4483.4 (1506.3)	0.338
	Potassium (mg/day)	2557.4 (1039.6)	2709.8 (1016.6)	0.202
	Calcium (mg/day)	604.8 (279.3)	613.1 (261.3)	0.788
	Magnesium (mg/day)	253.7 (94.4)	268.8 (94.7)	0.175
	Phosphorus (mg/day)	1124.6 (434.7)	1163.6 (436.6)	0.447
	Iron (mg/day)	7.8 (3.1)	8.1 (3.1)	0.382
	Zinc (mg/day)	8.1 (2.7)	8.4 (2.8)	0.400
	Copper (mg/day)	1.1 (0.4)	1.2 (0.4)	0.717
	Manganese (mg/day)	3.2 (1.1)	3.2 (1.1)	0.825
Fat-soluble vitamin	α-Carotene (μg/day)	465.4 (440.0)	421.1 (328.5)	0.261
	β-Carotene (μg/day)	3165.3 (2648.5)	3098.7 (2036.6)	0.784
	Retinol (μg/day)	474.5 (502.9)	444.1 (404.2)	0.527
	Cryptoxanthin (μg/day)	448.7 (400.0)	505.7 (402.8)	0.228
	β-Carotene equivalents (μg/day)	3627.0 (2919.5)	3567 (2246.2)	0.823
	Retinol equivalents (μg/day)	780.5 (587.9)	745.2 (485.7)	0.542
	Vitamin D (μg/day)	21.3 (14.6)	20.8 (14.3)	0.770
	α-Tocopherol (mg/day)	7.2 (3.1)	7.9 (3.1)	**0.049**
	β-Tocopherol (mg/day)	0.3 (0.1)	0.4 (0.1)	**0.004**
	γ-Tocopherol (mg/day)	11.0 (4.7)	12.7 (5.1)	**0.003**
	δ-Tocopherol (mg/day)	2.8 (1.1)	3.1 (1.2)	**0.033**
	Vitamin K (μg/day)	256.1 (165.1)	269.5 (165.2)	0.489
Water-soluble vitamin	Vitamin B1 (mg/day)	0.8 (0.3)	0.8 (0.3)	0.143
	Vitamin B2 (mg/day)	1.3 (0.5)	1.4 (0.5)	0.438
	Niacin (mg/day)	17.6 (7.6)	19.2 (7.7)	0.077
	Vitamin B6 (mg/day)	1.3 (0.5)	1.4 (0.5)	0.308
	Vitamin B12 (μg/day)	12.6 (7.8)	12.8 (7.8)	0.844
	Folic acid (μg/day)	330.3 (154.9)	348.5 (145.6)	0.287
	Pantothenic acid (mg/day)	6.6 (2.4)	6.8 (2.4)	0.573
	Vitamin C (mg/day)	125.6 (65.7)	135.2 (69.2)	0.235
Fatty acid	Saturated fatty acid (g/day)	13.6 (5.5)	14.7 (5.8)	0.105
	Monounsaturated fatty acid (g/day)	17.6 (7.2)	19.8 (7.6)	**0.016**
	Polyunsaturated fatty acid (g/day)	12.0 (4.5)	13.4 (5.1)	**0.014**
	n-3 fatty acid (g/day)	2.9 (1.4)	3.1 (1.5)	0.333
	n-6 fatty acid (g/day)	9.0 (3.3)	10.3 (3.8)	**0.004**
Others	Cholesterol (mg/day)	420.0 (199.7)	436.7 (200.6)	0.479
	Soluble dietary fiber (g/day)	2.9 (1.3)	3.1 (1.3)	0.194
	Insoluble dietary fiber (g/day)	8.5 (3.4)	8.9 (3.4)	0.241
	Dietary fiber (g/day)	11.8 (4.9)	12.4 (4.9)	0.319
	Salt equivalent (g/day)	10.9 (3.5)	11.3 (3.8)	0.341
	Sucrose (g/day)	13.9 (9.2)	14.2 (9.8)	0.845
	Alcohol (g/day)	5.4 (14.9)	8.7 (18.9)	0.127
	Daidzein (mg/day)	12.5 (9.0)	12.4 (9.3)	0.987
	Genistein (mg/day)	21.2 (15.3)	21.2 (15.8)	0.986

Values are means (standard deviation). * Student’s *t*-test, and *p* < 0.05 shown as boldface.

**Table 3:** Prevalence of sarcopenia according to the quantile of the intakes of vitamin E and fats.

Nutrient Factor	Characteristic	Daily Intake				Trend *p* *
		Q1 (Low)	Q2	Q3	Q4 (High)	
Fat	Median (range) of intake (g/day) ^†^	33.8 (6.8–41.4)	47.5 (41.5–53.4)	59.2 (53.5–66.7)	77.9 (66.7–143.8)	
	N with or without sarcopenia ^‡^	23/313	21/316	22/314	11/325	
	Adjusted OR (95% CI) ^§^	1.00 (reference)	1.01 (0.45–2.27)	0.75 (0.31–1.82)	**0.27 (0.08–0.96)**	**0.037**
Vegetable fat	Median (range) of intake (g/day) ^†^	16.5 (1.9–20.5)	24.1 (20.5–27.1)	30.2 (27.1–34.6)	40.0 (34.6–75.8)	
	N with or without sarcopenia ^‡^	27/309	20/317	17/319	13/323	
	Adjusted OR (95% CI) ^§^	1.00 (reference)	0.86 (0.40–1.83)	0.67 (0.28–1.58)	0.53 (0.18–1.50)	0.202
α-Tocopherol	Median (range) intake (mg/day) ^†^	4.6 (0.9–5.7)	6.6 (5.7–7.5)	8.4 (7.5–9.6)	11.2 (9.6–21.8)	
	N with or without sarcopenia ^‡^	25/311	17/320	26/310	9/327	
	Adjusted OR (95% CI) ^§^	1.00 (reference)	0.68 (0.30–1.55)	0.92 (0.38–2.19)	**0.14 (0.04–0.49)**	**0.008**
β-Tocopherol	Median (range) intake (mg/day) ^†^	0.21 (0.02–0.27)	0.31 (0.27–0.35)	0.39 (0.35–0.44)	0.51 (0.44–0.95)	
	N with or without sarcopenia ^‡^	24/312	24/312	22/315	7/329	
	Adjusted OR (95% CI) ^§^	1.00 (reference)	0.93 (0.44–1.94)	0.78 (0.34–1.78)	**0.24 (0.07–0.78)**	**0.018**
γ-Tocopherol	Median (range) intake (mg/day) ^†^	7.2 (0.9–9.2)	10.6 (9.2–12.0)	13.7 (12.0–15.4)	18.2 (15.4–36.1)	
	N with or without sarcopenia ^‡^	25/311	22/314	23/314	7/329	
	Adjusted OR (95% CI) ^§^	1.00 (reference)	1.31 (0.62–2.77)	1.07 (0.49–2.38)	**0.28 (0.09–0.87)**	**0.024**
δ-Tocopherol	Median (range) intake (mg/day) ^†^	1.7 (0.3–2.2)	2.6 (2.2–2.9)	3.3 (2.9–3.8)	4.4 (3.8–8.5)	
	N with or without sarcopenia ^‡^	22/314	24/313	20/316	11/325	
	Adjusted OR (95% CI) ^§^	1.00 (reference)	1.60 (0.75–3.42)	1.09 (0.47–2.55)	0.75 (0.26–2.12)	0.409
Monounsaturated fatty acid	Median (range) intake (g/day) ^†^	11.8 (2.0–14.5)	16.5 (14.6–18.8)	20.9 (18.8–24.0)	28.0 (24.0–53.3)	
	N with or without sarcopenia ^‡^	25/311	22/315	19/317	11/325	
	Adjusted OR (95% CI) ^§^	1.00 (reference)	0.94 (0.43–2.05)	0.69 (0.29–1.67)	**0.22 (0.07–0.72)**	**0.011**
Polyunsaturated fatty acid	Median (range) intake (g/day) ^†^	8.0 (1.6–9.9)	11.4 (10.0–12.7)	14.2 (12.7–16.0)	18.9 (16.0–34.1)	
	N with or without sarcopenia ^‡^	23/313	22/314	22/315	10/326	
	Adjusted OR (95% CI) ^§^	1.00 (reference)	1.19 (0.54–2.63)	0.96 (0.41–2.23)	**0.28 (0.09–0.89)**	**0.029**
n-6 fatty acid	Median (range) intake (g/day) ^†^	6.2 (0.9–7.6)	8.7 (7.6–9.8)	10.9 (9.8–12.2)	14.3 (12.2–26.3)	
	N with or without sarcopenia ^‡^	23/313	26/310	20/317	8/328	
	Adjusted OR (95% CI) ^§^	1.00 (reference)	1.65 (0.77–3.51)	1.01 (0.42–2.39)	0.39 (0.12–1.26)	0.064

OR: odds ratio, CI: confidence interval, Q: quantile. * Based on logistic regression analysis according to the quantile categories of nutrient intakes. *p* for trend estimated by treating the quantiles as ordinal variables for nutrient intakes. ^†^ Values at baseline. ^‡^ The number of subjects with or without sarcopenia. ^§^ Adjusted for age, sex, BMI, residence area, current smoking habit, current alcohol drinking habit, and total caloric intake. *p* < 0.05 shown as boldface.

## Data Availability

The data are not publicly available due to privacy. The data presented in this study are available in this text.
